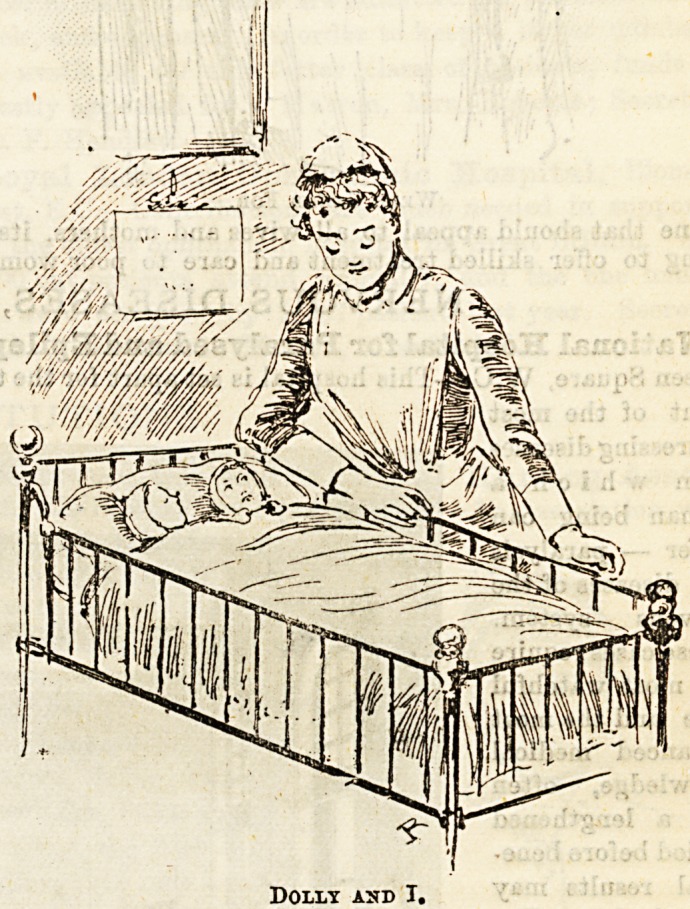# Hospitals for Children

**Published:** 1891-01-03

**Authors:** 


### HOSPITALS FOR CHILDREN.

**Hospital for Sick Children,** Great Ormond Street, W.C.—This was the first hospital in the kingdom specially designed for, and devoted to, the reception of sick children. It began in 1852 with 20 beds; this number has been now increased to 127, but still is insufficient. So many children urgently desire admission that, although upwards of 1,000 out-patients are treated every week, the Committee have been compelled to enlarge their space by building a new ward. The objects of the institution are the medical and surgical treatment of poor children, the wider extension and attainment of knowledge concerning their diseases, and the training of nurses for them. No one could visit this hospital and not feel that the saddest of all sights is a suffering child, suffering through no fault of its own, and enduring its pain with childish resignation. Visitors cannot fail to remember, when they see these little sufferers, surrounded with everything that skill, care, and thought can do to relieve them, what a contrast their present position presents to that of those left in their wretched homes to fight the disease as best they may. Surely many will be glad of the opportunity of contributing to so beneficent a work. Secretary, Mr. Adrian Hope, Great Ormond Street, W. C.; Matron, Miss Close.

On the Road to Recovery.

**Bristol Hospital for Sick Children and Women.**—This hospital was established in 1866, thanks to the devoted liberality of its present treasurer, Mr. Mark Whitwell, who displays a whole-hearted devotion to the interests of this charity. No expense has been spared to make the hospital efficient and attractive. It is supported entirely by voluntary contributions and donations, and annual subscriptions are earnestly solicited. No note of recommendation is required; children are admitted as in-patients free, and women on payment according to their means; and the out-patient department is on the provident principle. Eight hundred and thirty-two children and 56 women were received last year as in-patients, and 2,889 children, and 911 women as out-patients. In connection with the hospital are detached buildings for the isolation of infectious cases. Secretary, Mr. H. Lawford Jones; Matron, Miss Combe.

Dolly and I.

## Figures and Tables

**Figure f1:**
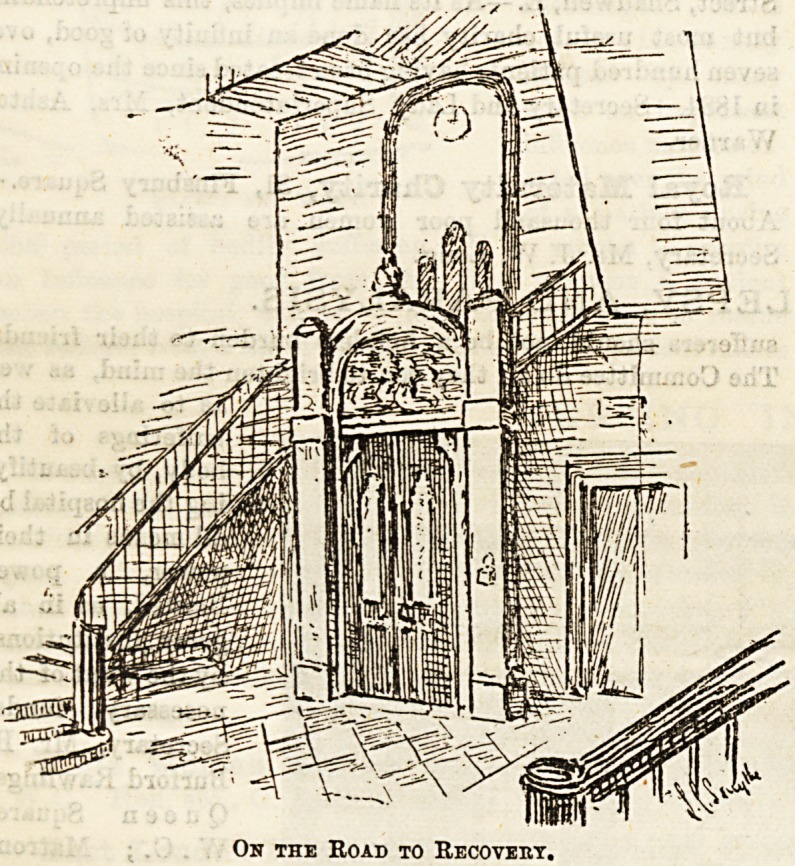


**Figure f2:**